# Emotional distress in cancer patients: the Edinburgh Cancer Centre symptom study

**DOI:** 10.1038/sj.bjc.6603626

**Published:** 2007-02-20

**Authors:** V Strong, R Waters, C Hibberd, R Rush, A Cargill, D Storey, J Walker, L Wall, M Fallon, M Sharpe

**Affiliations:** 1 School of Molecular and Clinical Medicine, Cancer Research UK and The University of Edinburgh. The University of Edinburgh Cancer Research Centre, The Western General Hospital, Crewe Road, Edinburgh EH4 2XR, UK; 2 Nuffield Department of Clinical Medicine, University of Oxford, Centre for Statistics in Medicine, Wolfson College Annexe, Linton Road, Oxford OX2 6UD, UK; 3 School of Molecular and Clinical Medicine, The University of Edinburgh Cancer Research Centre, The Western General Hospital, Crewe Road, Edinburgh EH4 2XR, UK; 4 Department of Statistics, Centre for Integrated Healthcare Research, Queen Margaret University College, Clerwood Terrace, Edinburgh EH12 8TS, UK; 5 Department of Statistics, SAS, Whittington House, Medmenham, Buckinghamshire, SL7 2EB, UK; 6 Department of Oncology, The Western General Hospital, Crewe Road, Edinburgh EH4, UK; 7 School of Molecular and Clinical Medicine, University of Edinburgh, Kennedy Tower, Royal Edinburgh Hospital, Morningside Park, Edinburgh EH10 5HF, UK; 8 Department of Oncology, Edinburgh Cancer Centre, Western General Hospital, Crewe Road, Edinburgh EH4 2XU, UK; 9 Department of Oncology, Edinburgh Cancer Centre, Western General Hospital, Crewe Road, Edinburgh EH4 2XU, UK; 10 School of Molecular and Clinical Medicine, The University of Edinburgh, Kennedy Tower, Royal Edinburgh Hospital, Morningside Park, Edinburgh EH10 5HF, UK

**Keywords:** distress, HADS, screening, prevalence, associations

## Abstract

To: (1) estimate the prevalence of clinically significant emotional distress in patients attending a cancer outpatient department and (2) determine the associations between distress and demographic and clinical variables, we conducted a survey of outpatients attending selected clinics of a regional cancer centre in Edinburgh, UK. Patients completed the Hospital Anxiety and Depression Scale (HADS) on touch-screen computers and the scores were linked to clinical variables on the hospital database. Nearly one quarter of the cancer outpatients 674 out of 3071 (22%; 95% confidence interval (CI) 20–23%) met our criterion for clinically significant emotional distress (total HADS score 15 or more). Univariate analysis identified the following statistically significant associations: age <65, female gender, cancer type and extent of disease. Multivariate analysis indicated that age <65 (odds ratio 1.41; 95% CI 1.18–1.69), female gender (odds ratio 1.58; 95% CI 1.31–1.92) and active disease (odds ratio 1.72; 95% CI 1.43–2.05) but not cancer diagnosis, were the independent predictors of clinically significant emotional distress. Services to treat distress in cancer patients should be organised to target patients by characteristics other than their cancer diagnosis.

Emotional distress refers to a continuum of psychological symptoms varying in severity (Carlson and Bultz, 2003). Reported prevalence rates of clinically significant emotional distress, defined here as cases of depression and anxiety, in cancer outpatients have varied from 15 to 42% (see Table 1). Despite the large number of studies published, we still have only limited information about the risk factors for clinically significant emotional distress in outpatients attending cancer centres. This is because the majority of published studies have either been small or of patients with specific cancer types. Of the published studies of outpatient samples with mixed cancer types only two have specifically reported the associations of clinically significant emotional distress in samples of more than 500 patients (Pascoe *et al*, 2000; Zabora *et al*, 2001) and none have studied patients attending clinics serving a geographically defined population. More data is therefore needed to best target resources for the management of emotional distress in cancer centres.

We therefore aimed to measure the prevalence of clinically significant emotional distress and to determine its demographic and clinical associations in a large sample of outpatients with a variety of cancer types attending a regional cancer centre.

## MATERIALS AND METHODS

### Setting

The study took place in the Edinburgh Cancer Centre, which is a regional, tertiary, cancer centre and is the sole provider for specialist cancer services to a geographically defined population of approximately 1.5 million people in the South East of Scotland UK.

### Sample

We included consecutive follow-up attenders over the age of 18 at the following diagnosis based cancer clinics: colorectal, breast, gynaecological, genitourinary, sarcoma, melanoma and mixed cancers (but not lung, upper gastrointestinal, head and neck and haematological cancer services as the screening system was not operating in these clinics). We excluded patients who were attending the cancer centre for the first time, those screened within the previous month and also those who were unable to respond because of being too ill, unable to read English or who had major communication or cognitive problems. Recruitment took place over 18 months from June 2003 to December 2004.

### Design

Cross-sectional survey linking self-report and clinical data.

### Procedure

A semiautomated symptom screening service had been established in the clinic in order to provide clinical information on patients' physical and psychological symptoms to their cancer team. As part of this system all individuals attending follow-up outpatient clinics were invited to complete the Hospital Anxiety and Depression Scale (HADS) (Zigmond and Snaith, 1983). After the patient had checked in at reception, the questionnaire was administered on touch-screen computers situated in a dedicated suite adjacent to the consultation rooms, and the results were made available to the Oncologist before the consultation. The use of computers in screening for quality of life and psychological distress in patients with cancer has been found to be an acceptable and efficient way to obtain self-report information (Allenby *et al*, 2002).

### Ethical approval

As the data were collected as part of the clinical service individual patient's consent was not obtained. Approval for the aggregated anonymised data to be reported was obtained from the local research ethics committee.

### Measures

Emotional distress was measured using the HADS. This scale was chosen over others because it is well established, widely used, acceptable to patients and has been extensively used in both cancer and non-cancer patients. The HADS is a self-rated 14-item questionnaire specifically designed for patients with medical illness. It has depression and anxiety subscales with seven items each. These two subscales correlate highly and HADS scores are frequently analysed as a single scale (Bjelland *et al*, 2002). Individual items are rated on a four-point scale (0–3), resulting in maximum scores of 21 on each subscale and a total maximum score of 42. Patients are asked to report symptoms over the previous week.

Clinically significant emotional distress was defined as a total HADS score of 15 or above. This cutoff score was reported by Ibbotson *et al* (1994) to be the best for identifying patients likely to have an interview based diagnosis of depressive or anxiety disorder. The reliability, validity and factor structure of the HADS has been established in a variety of clinical populations (Moorey *et al*, 1991; Johnston *et al*, 2000; Mykletun *et al*, 2001; Smith *et al*, 2002) and validated in this population of cancer patients. We found that a cutoff of 15 or above on the total HADS score gave a sensitivity of 0.87 (95% confidence interval (CI) 0.70–0.95), a specificity of 0.85 (95% CI 0.81–0.89) and a positive predictive value of 0.35 for Major Depressive Disorder (Walker *et al*, in press). We also analysed the depression and anxiety subscales separately using the recommended cutoff scores (Bjelland *et al*, 2002) of nine or more on the anxiety subscale and eight or more on the depression subscale.

The cancer centre clinical database contained data on patients' demographic and clinical characteristics including cancer type, clinical staging of disease and treatment received. The patients' clinical data relevant to the time of the selected screening event was anonymised and matched to the HADS score using a unique patient identification number and date of birth. The primary cancer type was classified according to the site of origin. In cases with more than one cancer type, the cancer that was dominating treatment at the time of screening was recorded. Disease status was classified into ‘disease free’ and ‘active disease’. Treatment status was determined by identifying the treatment the patient had received within 2 months before the screening date and categorised as ‘no anti-cancer treatment’, ‘receiving hormone treatment’ or ‘receiving chemotherapy and/or radiotherapy treatment’ (see Appendix A-online). The accuracy of the data recorded on the electronic database was checked against patients' paper case notes in a 5% random sample and good agreement (97%) found.

### Analysis

The statistical analysis first compared the characteristics of eligible patients on whom we had complete data with those patients with missing HADS data or who had refused screening in order to assess to what extent the sample was representative of the eligible population. The HADS total and depression and anxiety subscale scores were described by calculating the medians and interquartile ranges.

The prevalence of clinically significant emotional distress, and depression and anxiety separately (and the 95% CI around these estimates) were calculated using the cutoff scores described above. The associations of clinically significant emotional distress with cancer type, extent of disease, treatment, age and gender were then examined using univariate logistic regression. Multivariate logistic regression, using the method of stepwise selection, was subsequently applied to identify independent predictors of clinically significant emotional distress.

All statistical analysis were carried out using SAS version 9.1 software, with Stata version 9 for calculating the CI for prevalence estimates.

## RESULTS

### Sample

**Figure 1:**
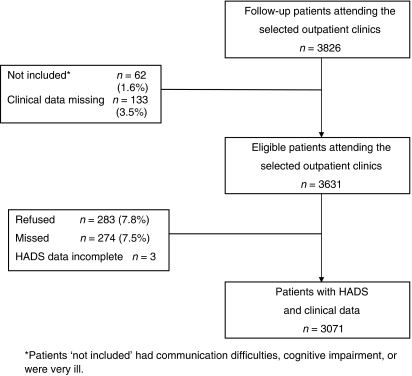
Flow diagram of patients surveyed indicating derivation of final sample.

Data were available on 3071 patients, representing 85% of eligible clinic attendees. The details of how the final sample was derived and the reasons for missing data are shown in Figure 1.

Table 2 shows the cancer characteristics of the final sample together with those for the patients whose data were missing or incomplete. There were modest differences between these groups in all variables other than treatment received. These differences, especially disease severity, mainly reflect the difficulty obtaining screening data from very ill patients. The large number of patients classified as disease-free, reflects the number attending for post treatment follow-up.

### HADS score

**Figure 2:**
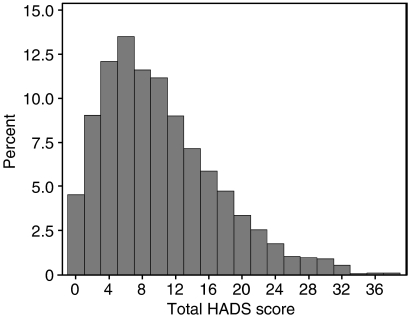
Distribution of total HADS scores of sample (*n*=3071).

The distribution of the total HADS scores is shown in Figure 2. As can be seen, the distribution is skewed towards lower scores (less distress).

The median scores for the HADS total score and the two subscales together with prevalence rates and 95% CIs are shown in Table 3.

Using univariate analysis, we examined associations between cases of clinically significant emotional distress and the pre-stated demographic variables and cancer characteristics (Table 4). This analysis indicated that patients, who were female, had active disease and were aged <65 were more likely to be cases. There was also an association with cancer type.

Multivariate logistic regression analysis was used to identify the most important independent predictors of clinically significant emotional distress and the results are shown in Table 5. Having accounted for the effect of age, gender and extent of disease, no other factors emerged as significant predictors. Being female and having active disease both increase the likelihood of distress, whereas being over 65 reduces the likelihood. Notably cancer type was not a predictor.

The associations with clinically significant anxiety and depression were similar to those with clinically significant emotional distress (depression or anxiety). Only gender and extent of disease were independent predictors for cases of clinically significant depression whereas age was also a predictor for anxiety.

## DISCUSSION

### Main finding

Almost a quarter (22%; 95% CI 20–23%) of our sample of outpatients at a cancer centre attending colorectal, breast, gynaecological, genitourinary, sarcoma, melanoma and mixed cancer clinics had clinically significant emotional distress defined as a total HADS score of 15 or more. Furthermore, these cases were not uniformly distributed in the sample; independent predictors of distress were being female, having active disease, and being aged <65. The type of cancer was associated with distress in the univariate analysis but did not emerge as an independent predictor in the multivariate analysis.

### Limitations

These findings must be set in the context of a number of limitations. The first category concerns the patient sample: (a) we did not survey all the clinics in the cancer centre. Several cancer clinics including lung, upper gastrointestinal, head and neck and haematological cancers were not included. As other studies have reported a high prevalence of emotional distress in patients with these cancers (Zabora *et al*, 2001; Montgomery *et al*, 2003), the findings presented may have underestimated the prevalence of clinically significant emotional distress in the whole cancer centre; (b) not all patients attending the cancer centre completed the HADS screening. New outpatient attenders were excluded at the request of the clinicians. In addition, a number of patients did not complete the screening for reasons previously detailed and there were modest but statistically significant differences between the analysed sample and those on whom we had incomplete data. The patients who did not complete the screening were more likely to be younger, male, with testicular cancer or patients with advanced disease. Despite these limitations, this study is the largest survey of clinically significant emotional distress and its associations yet conducted in a sample of mixed cancer outpatients referable to a geographically defined population.

The second category of limitations concerns the measures used: our definition of ‘clinically significant emotional distress’ was based on a self-rated questionnaire and not on a clinical interview. This means that many patients with transient distress, who would not receive duration-based diagnoses from a clinical interview, will have been included. A diagnostic interview that interrogated patients about the timing of symptoms would be expected to produce a lower prevalence estimate of cases. There has also been some controversy about the validity of the HADS at the recommended cutoff scores in detecting distress in patients with all cancer types and at all disease stages (Ibbotson *et al*, 1994; Hall *et al*, 1999). The scores we used were based on the best available data and on our own validation study. Furthermore, the use of cutoff scores allowed us to estimate the actual prevalence of clinically significant emotional distress and not just mean values in the sample.

### Relationship of findings to other studies

There have been few studies with which our findings can be compared directly because of the variety of measures and criteria that have been used (a list is given in Table 1) and the range of populations studied. Some comparison can be made with the two large studies of outpatients with mixed cancers that reported the prevalence of ‘cases’ of distress and its associations. Pascoe *et al* (2000) in an Australian study used the HADS at a lower cutoff for clinically significant distress and higher cutoffs for anxiety and depression and found comparable prevalence rates of 31% for distress, 12% anxiety and 7% depression. Zabora *et al* (2001) in a study from the US used the Brief Symptom Inventory, and reported case prevalence rates of 35% for distress, 24% anxiety and 19% depression. Pascoe *et al* (2000) found that being female, aged <65 years and having a reduced activity status (which may be regarded as measuring a similar concept to advanced disease) were associated with distress, whereas Zabora *et al* (2001) found younger age and lower income to be associated with higher levels of distress but did not find an association with gender. Neither found a strong association with disease type. The results reported here confirm that in a large sample from a geographically defined population from the UK cancer type is not an important predictor of emotional distress and that female gender, younger age and severity of disease are.

### Implications

Despite the large number of studies that have been published studying emotional distress in cancer patients, general conclusions have been difficult to draw because of their methodological limitations and diverse measures. It would be helpful if future studies adopted similar measures and agreed cutoff scores for clinical significance to allow meaningful comparison between them.

The findings of this survey highlight the prevalence of clinically significant emotional distress in an outpatient cancer population and consequently the need for services to attend to this. Although some diagnosis-based cancer services will have a higher prevalence of emotional distress than others, the analysis of independent predictors implies that if efforts to identify cases are to be targeted, variables other than cancer type are likely to be most useful. General cancer centre based psychological services may be more efficient than diagnosis based ones.

## CONCLUSION

The results of this study emphasise the need to develop services to improve the management of emotional distress in outpatient cancer services and suggest how these may be best targeted. Further studies are now required to design and test appropriate therapeutic interventions for patients who have been identified as having clinically significant emotional distress.

## Figures and Tables

**Table 1:** Studies of prevalence of clinically significant emotional distress and its associations in cancer outpatients

**Authors and year of publication (country)**	**Sample characteristics (*n*)**	**Self-rated measure^a^ (cutoff used)**	**General distress**	**Anxiety**	**Depression**	**Examined clinical associations (yes/no)**
Balderson and Towell 2003 (UK)	Prostate (94)	HADS (total ⩾15)	38%			y
Berard *et al* 1998 (S Africa)	Breast, head and neck, lymphoma (456)	HADS (D⩾8)			14%	n
Bisson *et al* 2002 (UK)	Prostate (88)	HADS (A ⩾11, D⩾11)		8%	0%	y
Bradley *et al* 2005 (Canada)	Advanced mixed cancers, receiving radiotherapy (1296)	ESAS (total ⩾4)		29%	25%	y
Dahl *et al* 2005 (Norway)	Testicular (1408)	HADS (A ⩾8, D ⩾8)		19.2%	9.7%	y
Fallowfield *et al* 2001 (UK)	Mixed cancers (2297)	GHQ12 (total ⩾4)	36.4%			n
Ford *et al* 1995 (UK)	Mixed cancers, newly diagnosed (117)	GHQ-30 (cutoff not specified)	30%			n
		HADS (A ⩾11, D⩾11)		26%	7%	
Grassi *et al* 2004 (Italy)	Mixed cancers (227)	HADS (A⩾11, D⩾11)		17%	9%	n
Hahn *et al* 2004 (USA)	Mixed cancers receiving radiotherapy (124)	BDI-II (total ⩾14)	15%			n
Hopwood *et al* 1991 (UK)	Advanced Breast cancer (81)	HADS (A⩾11 or D⩾11)	42%			n
Ibbotson *et al* 1994 (UK)	Mixed cancers (513)	HADS (total ⩾15)	17%			n
Norton *et al* 2004 (USA)	Ovarian, mostly advanced disease (143)	BDI (cutoff not specified)	20%			y
Pascoe *et al* 2000 (Australia)	Mixed cancers (504)	HADS (A ⩾11, D⩾11, total ⩾8)	30.6%	11.5%	7.1%	y
Razavi *et al* 1992 (Belgium)	Lymphoma (117)	(self-rated) DSM II-R	36.8%			n
Sharpe *et al* 2004 (UK)	Mixed cancers (3938)	HADS (total ⩾ 15)	23%			n
Yan and Sellick 2004 (China)	Gastro-intestinal, newly diagnosed on active treatment (146)	BDI-13 (total ⩾5)			27.4%	y
Zabora *et al* 2001 (USA)	Mixed cancers (4496)	BSI (global severity index or total symptom score ⩾63 on either BSI subscale)	35%	24.1%	18.7%	y

BDI-II and BDI-13 variations on the Beck Depression Inventory; BSI=Brief Symptom Inventory;

a BD1, ESAS=Edmonton Symptom Assessment Scale; GHQ-30 and is the General Health Questionnaire–GHQ-30 items; HADS=Hospital Anxiety and Depression Scale and 12 items.

DSMII-R is interview is the Structured Clinical Interviews for DSM Axis 1 Disorders; n=no; y=yes. A=anxiety subscale; D=depression subscale.

**Table 2:** Demographic and clinical characteristics of the eligible patients with complete data and those with incomplete data (*n*=3631). Numbers shown are percentages (*n*) except when specified

**Variable**	**Complete data**	**Incomplete data**	**P-value^a^**
*Total*	3071	560	
*Age (continuous)*			0.0113
Median (range)	62.0 (18.2 to 93.1)	63.5 (21.5–92.9)	
			
*Age (categorical)*			0.0149
<65	58 (1793)	53 (296)	
⩾65	42 (1278)	47 (264)	
			
*Gender*			0.0016
Male	34 (1048)	41 (230)	
Female	66 (2023)	59 (330)	
			
*Cancer type*			0.0004
Breast	35 (1084)	31 (172)	
Bowel	15 (458)	14 (81)	
Prostate	12 (359)	12 (68)	
Ovarian	10 (305)	8 (43)	
Other gynaecological	10 (313)	9 (48)	
Testicular	8 (247)	13 (70)	
Other^b^	10 (305)	14 (78)	
			
*Extent of disease*			0.0049
Disease free	67 (2068)	61 (343)	
Active disease	33 (1002)	39 (217)	
Unknown^c^	0.03 (1)		
			
*Treatment*			0.0861
No anti-cancer treatment	55 (1684)	58 (326)	
Hormone	17 (518)	18 (101)	
Chemo and/or radiotherapy	28 (869)	24 (133)	

a Except for age (continuous), all *P*-values are from a chi-square test. Age (continuous) is compared using the Mann–Whitney *U*-test. The number of unknown records is not included as part of the chi-square test.

b The group ‘other’ contained the following cancers: lung, *n*=82; melanoma, *n*=63; sarcoma, *n*=55; kidney, *n*=28; primary peritoneal, *n*=18; bladder, *n*=14; head and neck, *n* =11; upper GI, *n*=6; pancreatobiliary, *n* =6; haematology, *n*=3; penis, *n*=3; adrenal, *n*=2; epididymis, *n* =1; and ‘unknown primary cancer, *n*=13.

c Insufficient clinical data available to determine extent of disease.

**Table 3:** HADS scores (*n*=3071)

**Scale**	**Median (range)**	**cutoff criterion**	**Sample prevalence Percent (number)**	**Population prevalence estimate 95% Confidence interval**
Total HADS	8 (0–38)	⩾15	22 (674)	20–23
Anxiety subscale	5 (0–21)	⩾9	23 (704)	21–24
Depression subscale	3 (0–21)	⩾8	16 (482)	14–17

HADS=Hospital Anxiety and Depression Scale.

**Table 4:** Univariate analysis association with clinically significant emotional distress, anxiety and depression with demographic and clinical variables (*n*=3071). Numbers shown are percentages (*n*) except when specified

**Variable**	**Anxiety (score ⩾9) % (*n*)**	**Depression (score ⩾8) % (*n*)**	**Distress (total score ⩾15) % (*n*)**	**Odds ratio for distress (95% CI)**	***P*-value**
*Age (categorical)*					0.0007
<65	28 (493)	15 (273)	24 (432)	1.00	
⩾65	17 (211)	16 (209)	19 (242)	0.74 (0.62–0.88)	
*Gender*					<0.0001
Male	16 (168)	12 (124)	17 (177)	1.00	
Female	27 (536)	18 (358)	25 (497)	1.60 (1.32–1.94)	
					
*Cancer type*					<0.0001
Breast	26 (283)	18 (192)	23 (252)	1.22 (0.93–1.6)	
Bowel	16 (71)	14 (62)	20 (91)	1.00	
Prostate	14 (49)	13 (46)	15 (53)	0.70 (0.48–1.01)	
Ovarian	30 (92)	18 (54)	27 (83)	1.51 (1.07–2.12)	
Other gynaecological	27 (85)	15 (47)	23 (72)	1.21 (0.85–1.71)	
Testicular	18 (45)	7 (18)	16 (39)	0.76 (0.50–1.14)	
Other	26 (79)	21 (63)	28 (84)	1.53 (1.09–2.15)	
					
*Extent of disease*					<0.0001
Disease free	21 (443)	13 (264)	19 (395)	1.00	
Active disease	26 (260)	22 (217)	28 (278)	1.63 (1.36–1.94)	
					
*Treatment*					0.0716
No anti-cancer treatment	22 (366)	14 (230)	21 (346)	1.00	
Hormone	27 (138)	19 (99)	25 (130)	1.30 (1.03–1.63)	
Chemo and/or radiotherapy	23 (200)	18 (153)	23 (198)	1.14 (0.94–1.39)	

**Table 5:** Multivariate analysis for independent predictors of clinically significant emotional distress (*n*=3071)

**Variable**	**Distressed % (*n*)**	**Not distressed % (*n*)**	**Odds ratio (95% CI)**	***P*-value**
*Age (categorical)*				0.0002
<65	24 (431)	76 (1361)	1.00	
⩾65	19 (242)	81 (1036)	0.71 (0.59–0.85)	
				
*Gender*				<0.0001
Male	17 (176)	83 (871)	1.00	
Female	25 (497)	75 (1526)	1.58 (1.31–1.92)	
				
*Extent of disease*				<0.0001
Disease-free	19 (395)	81 (1673)	1.00	
Active disease	28 (278)	72 (724)	1.72 (1.43–2.05)	

## Appendix A

### CANCER AND TREATMENT STATUS CLASSIFICATIONS

#### Cancer diagnosis

Owing to the small numbers of patients in some categories, the primary cancer types were grouped into seven major categories: breast, testicular, ovarian, prostate, bowel (included rectal, colon and anal sites of origin), other gynaecological (included cervical, uterine, vulva and vaginal sites of origin), and others (included lymphoma, head and neck, lung, upper gastro-intestinal, melanoma, brain and central nervous system, kidney, adrenal gland, bladder, epididymis, sarcoma, primary peritoneal, basal cell and unknown primary cancers).

For the majority of the patients, their primary cancer type was classified according to the site of origin. The only exceptions were:

1. Melanoma. This was classified as melanoma regardless of the site of origin
2. Germ cell tumour in regions other than the gonads (e.g. mediastinal) was classified in men as testicular and in women as ovarian cancer.

Those patients who had more than one primary cancer type were classified according to the cancer they were being treated for at the time of screening. If they were being treated for more than one malignancy concurrently, they were classified according to the cancer that was dominating their treatment at the time of screening.

Those patients, who were disease-free and on no treatment for any of their previous cancers, were classified according to their most recently diagnosed cancer.

#### Cancer status at time of screening

Patients were classified according to their clinically detectable cancer status at the time of screening. The categories were ‘disease-free’ and ‘active disease’ or ‘unknown’. Some clinical situations could be classified in more than one way. For clarity, these are outlined under the appropriate section.

#### Disease-free

- Undergoing post surgical adjuvant radiotherapy/chemotherapy/hormone therapy with no clinically detectable residual disease.
- After completion of primary radical chemotherapy or radiotherapy given with curative intent and no documented residual disease or recurrence (i.e. early anal cancer after radical chemoradiation and stage 3 ovarian cancer after chemotherapy with normal serum markers)
- Metastatic cancer which had been surgically removed and no documentation of recurrence (e.g. liver metastases from bowel cancer, which were removed after chemotherapy and partial hepatectomy)

#### Active disease (local and metastatic disease categories combined)

##### Local disease

- Clinically detectable local disease or regional lymph node metastases
- Undergoing primary chemotherapy or radiotherapy with curative intent, for example, early anal cancer
- Undergoing neo-adjuvant chemotherapy/radiotherapy/hormone treatment before surgery (unless metastatic disease)

##### Metastatic

- Metastases to organs or distant lymph nodes
- Stage 3 ovarian cancer undergoing primary or post-surgical chemotherapy at the time of screening (because it is assumed there is residual disease in the abdominal cavity)
- Relapsed ovarian cancer
- Primary lung cancer with disease in more than one lobe of lung because it was not treated with curative intent
- Breast cancer with supra-clavicular nodes

##### Unknown

When it was not clear what the cancer status was at the time of screening

#### Treatment status at the time of screening

Treatment status was described according to what treatment had been received within the 2 calendar months before screening: no anti-cancer treatment, hormone treatment, chemotherapy, radiotherapy, surgery or any combination of these (although this does not automatically imply that they were concurrent). These were collapsed into the three groups presented in the paper: (1) no anti-cancer treatment (which included those who had had surgery); (2) hormone treatment and (3) chemotherapy and/or radiotherapy treatment. Those who had received two treatment modalities within the last 2 months were allotted to the most clinically dominant category according to the following ranking: chemo/radiotherapy >hormones >surgery, for example, those who had received both surgery and chemotherapy were assigned to the chemo and/or radiotherapy treatment group.

No distinction was made between the dose and fractionation of radiotherapy administered; patients were classed as undergoing radiotherapy provided at least one fraction of treatment had been administered within the previous 2 calendar months.

The arbitrary 2-month cutoff was made on the basis that patients' symptoms might still be affected by treatment such as chemotherapy, completed several weeks before screening. The authors recognise that possible treatment associated symptoms may resolve before or after this time.
